# Expert Recommendations for the Diagnosis, Treatment, and Management of Adult B-Cell Acute Lymphoblastic Leukemia in Latin America

**DOI:** 10.1200/GO.22.00292

**Published:** 2023-05-11

**Authors:** Ana Lisa Basquiera, Maria Cristina Seiwald, Carlos Roberto Best Aguilera, Leonardo Enciso, Isolda Fernandez, Angela Marie Jansen, Elenaide Nunes, Matias Sanchez del Villar, Victor I. Urbalejo Ceniceros, Vanderson Rocha

**Affiliations:** ^1^ Hematology and Oncology Service, Bone Marrow Transplant Program, Hospital Privado Universitario de Cordoba, Instituto Universitario de Ciencias Biomédicas de Cordoba (IUCBC), Cordoba, Argentina; ^2^ Department of Clinical Medicine, Hematology and Hemotherapy, University of Sao Paulo (FMUSP), Sao Paulo, Brazil; ^3^ Conacyt National Quality Postgraduate Program, University of Guadalajara & Western General Hospital, Guadalajara, Mexico; ^4^ National University of Colombia, Bogota, Colombia; ^5^ Department of Hematology, Fundaleu, Buenos Aires, Argentina; ^6^ Americas Health Foundation, Washington, DC; ^7^ Hospital de Clínicas—Federal University of Parana, Parana, Brazil; ^8^ Chief Bone Marrow Transplant Service, Department of Hematology, Clinica Alemana de Santiago, Santiago, Chile; ^9^ Department of Hematology, National Cancer Institute, Mexico City, Mexico

## Abstract

**PURPOSE:**

Despite strong induction chemotherapy response rates, only 30%-40% of patients with adult B-cell acute lymphoblastic leukemia (ALL) attain long-term remission. This study analyzes ALL in Latin America (LA) and recommends diagnosis, treatment, and management protocols.

**METHODS:**

The Americas Health Foundation organized a panel of hematologists from Argentina, Brazil, Chile, Colombia, and Mexico to examine ALL diagnosis and therapy and produce recommendations.

**RESULTS:**

Lack of regional data, unequal access to diagnosis and therapy, inadequate treatment response, and uneven health care distribution complicate adult ALL management. The panel recommended diagnosis, first-line and refractory treatment, and post-transplantation maintenance. Targeted treatments, including rituximab, blinatumomab, and inotuzumab ozogamicin, are becoming available in LA and must be equitably accessed.

**CONCLUSION:**

This review adapts global information on treating ALL to LA. Governments, the medical community, society, academia, industry, and patient advocates must work together to improve policies.

## INTRODUCTION

Acute lymphoblastic leukemia (ALL) is a heterogeneous malignant disease with a notable proliferation of immature lymphoid cells that invade the bone marrow, blood, and extramedullary sites.^1^ Although 80% of ALL occurs in children, it follows a bimodal distribution, with the second peak occurring around age 50 years, representing a devastating disease when it occurs in adults. Its epidemiology varies widely among populations, on the basis of ethnicity and other factors.

**CONTEXT**

**Key Objective**
This review provides a state-of-the-art series of recommendations for treating adult B-cell acute lymphoblastic leukemia in Latin America.
**Knowledge Generated**
This review, written by a panel of oncologists from Argentina, Brazil, Chile, Colombia, and Mexico, provides detailed first- and second-line treatment protocols for patients in the region.
**Relevance**
To advance targeted treatment and precision medicine, all stakeholders must collaborate to create and enforce policies that provide the best available care for patients.

Interestingly, Latin American populations have a lower incidence among all subtypes of leukemias except ALL, where Latin Americans have a higher incidence and a more dismal prognosis.^2^ Although there have been improved outcomes for pediatric patients, adult prognosis remains poor. In addition, adult patients with precursor B-cell ALL present with high-risk (HR) genetic alterations four times more often than pediatric patients. Despite high response rates to induction chemotherapy, only 30%-40% of adult patients achieve long-term remission.^3^ This review examines the landscape and challenges of ALL in Latin America (LA) and provides recommendations for its diagnosis and treatment tailored to the region. The recommendations are based on an extensive literature review and expert opinion to contribute to uniform patient care and improved outcomes.

## METHODS

The Americas Health Foundation (AHF) gathered nine ALL hematologists from Argentina, Brazil, Chile, Colombia, and Mexico. They held a 3-day conference on February 20-22, 2022, to generate recommendations for diagnosing, treating, and managing adult ALL in LA. The AHF researched ALL through PubMed, MEDLINE, and EMBASE. Treatment, diagnosis, management, and allogeneic hematopoietic stem-cell transplantation in combination with Latin America, Argentina, Brazil, Chile, Colombia, and Mexico were searched with dates ranging from January 1, 2016, until December 10, 2021. The articles identified were in English, Portuguese, and Spanish. The AHF supplemented this search by contacting thought leaders in the LA medical community to ensure that the list appropriately reflected the relevant areas. This manuscript's authors include all the specialists who attended the conference.

The AHF prepared questions to address barriers restricting access to ALL diagnosis and treatment in LA and allocated one to each panel member (Data Supplement). Panel members submitted written responses to their question on the basis of the literature and their knowledge. The panel examined and modified each narration during the 3-day meeting via multiple debate rounds. An AHF staff member moderated the debate. The panel unanimously endorsed the recommendations on the basis of the facts obtained, professional opinion, and personal experience. The completed paper was sent to the panel for evaluation and approval after the conference.

## RESULTS

### Epidemiology

There are scarce epidemiologic data on ALL in LA. Still, retrospective studies indicate that ALL may be more prevalent in people of LA descent than in the United States and Europe. The US's SEER Program reported a frequency of 18 cases per million for all ages and races. However, in the Latin population in the United States, the proportion increased significantly to 26.6 cases per million.^2^ A global analysis found the highest incidence of leukemia in Hispanic people, with 35 cases per million.^4^ Data suggest that the rate has been increasing rapidly over time and a worse prognosis was observed when compared with other ethnic groups. The underlying cause for this is unknown although several hypotheses exist, including socioeconomic disparities, environmental risks, and genetic variants.^3,5-8^

The lack of regional data on ALL reflects the methodology used in the public health registries, where data are either lacking or only provided as a histopathologic diagnosis. A study of over 1,000 adults in LA with leukemia diagnosed by multiparametric flow cytometry (MFC) found that 519 patients were diagnosed with leukemia (51%). Of those, 486 patients (93.6%) had B-cell lineage.^9^ Transplant data may support the hypothesis that ALL is more prevalent in LA. For instance, a comparison of patients undergoing allogeneic hematopoietic stem-cell transplants (allo-HSCT) because of ALL in LA (27%) was higher than those in Europe (16%).^10,11^ More regional data are crucial to determine the actual prevalence of ALL, understand the disease behavior, and improve regional patient outcomes.

### Access to Testing

Geography, natural resources, politics, development, racial and ethnic representation, education, wealth distribution, and health care are diverse in LA.^1^ 20%-66% of LA residents face health care barriers like long wait times, geographical distances, availability, accessibility, and convenience.^12^ In LA and the Caribbean, the percentage of the gross domestic product (GDP) invested in health care is less than half that of the US level.^13^

Access to adequate diagnosis and treatment of ALL is often challenging in LA. A survey conducted by the American Society for Hematology and LA Hematology societies showed that countries in LA have heterogeneous access, which varies according to whether the patient has public or private health insurance.^14^ Basic treatment is generally guaranteed. However, high-cost therapies and medications like monoclonal antibodies (MoAbs) and allo-HSCT^8^ are often inaccessible. In some cases, sponsored programs from the pharmaceutical industry fund these therapies, providing a short-term solution to access.^14^

### Initial Diagnosis

The diagnosis of ALL was based on 2016 WHO classification guidelines that integrate the characterization of cell morphology, immunophenotypes, genetics, and cytogenetics.^15^ Initial workup should include a thorough medical history, physical examination, laboratory assays, and imaging studies.^16^

Patients with Philadelphia-like ALL are highly heterogeneous, with a broad spectrum of genetic alterations requiring different identification approaches.^17^ Emerging data are appearing in LA in the pediatric population.^18^

### Prognosis and Risk Stratification

Identifying risk factors and stratification are needed to select appropriate treatment regimens and assess allo-HSCT eligibility.^1^ Although the suggested diagnostic workup permits the identification of some HR subsets, clinical risk groups are further determined by several disease- or host-related factors. Response dynamics refine prognoses. Patients presenting with no risk factors are defined as standard-risk (SR). Classic prognostic factors are shown in Table 1.^1,6,16,19^ However, some of these parameters have been identified, at least in part, as surrogate markers for other abnormalities, in particular, genetic alterations.

**TABLE 1:**
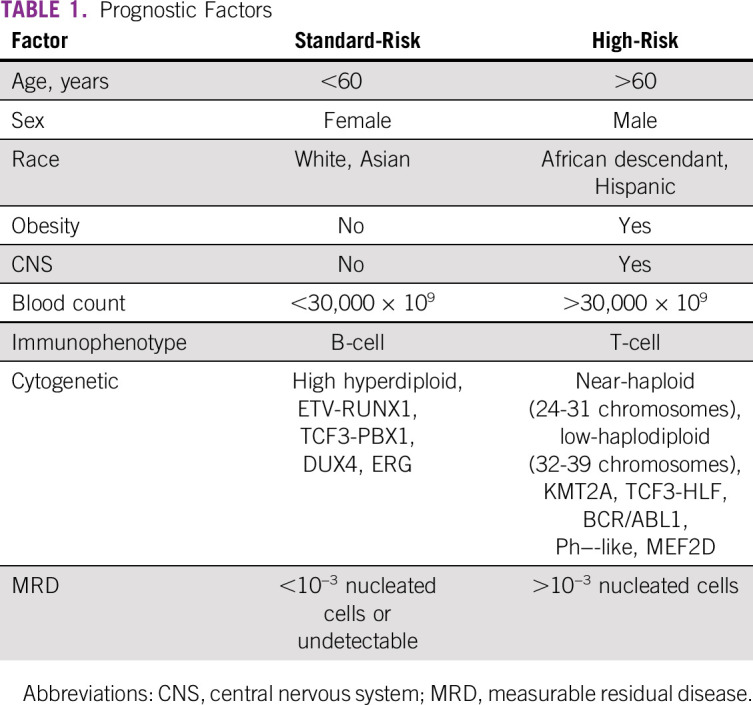
Prognostic Factors

Treatment response is recognized as a prognostic factor in ALL. The measurable residual disease (MRD), which is the presence of disease in patients in complete remission (CR) by conventional analysis, has become the standard measure for evaluating the prognostic impact of treatment. Flow cytometry and molecular methods can be used to detect MRD:quantitative polymerase chain reaction (qPCR) for immunoglobulin variable region heavy-chain gene rearrangements, qPCR for gene fusions, and next-generation sequencing can measure MRD, with a high correlation in results between molecular and immunophenotype assays.^20^ Multiparameter flow cytometry has been widely used because it is faster, relatively inexpensive, and sensitive. However, it requires significant technical expertise and standardization. Recognizing the benefits of the MFC, low-income countries have increased efforts for widespread adoption to allow for broader data reproducibility.

Current recommendations are to incorporate MRD in making therapeutic decisions, considering MRD+ after induction and in early consolidation a powerful predictor of relapse and low overall survival (OS).^21^ A result of positive MRD indicates treatment resistance and, thus, the need for allo-HSCT or targeted therapy.^18^ Only one targeted therapy, blinatumomab, is approved to eradicate MRD.^22^

### Response to Treatment in ALL

In general terms, adults' response to ALL treatment remains unsatisfactory. In pediatric patients, long-term remissions have reached 90% in some series, depending on risk factors. By contrast, adults have a 5-year OS of <45%. Patients older than 60 years are a particularly vulnerable group, not only because of the disease but also the frailty and comorbidities this group generally presents, with only 20% attaining 5-year OS.^23^

However, novel drugs such as tyrosine kinase inhibitors (TKI), MoAbs-directed against CD19, CD20, and CD22 and drugs interfering with the overexpression of genes involved in tumor survival offer opportunities to personalize therapy on the basis of clinical characteristics and organ fitness of the patient.^1^ With promising results, the transition toward personalized treatment appears to be underway. An intelligent combination of conventional chemotherapy with novel drugs has the potential to change the game in the treatment of ALL.

## TREATMENT RECOMMENDATIONS

The patient's age range (adolescents, adults, or elderly) must be considered when determining the best ALL treatment, and treatment goals differ accordingly. The first-line treatment typically includes four phases over 2-3 years: induction, consolidation, intensification, and long-term maintenance. In addition, directed treatment is given to treat or prevent central nervous system (CNS) disease. The protocol includes serial intensive intrathecal chemotherapy with methotrexate alone or methotrexate, cytarabine, and corticosteroids in conjunction with high-dose intravenous methotrexate and cytarabine. In select patients, cranial radiotherapy may be needed in the case of CNS leukemia (CNS-3, defined as white blood count ≥5/μL in cerebral spinal fluid with the presence of lymphoblasts and/or cranial nerve involvement).^21^ Several protocols are used in LA to treat adults with ALL.^24-34^ Results with first-line regimens used in LA for adult patients with B-cell precursor ALL are shown in Table 2.

**TABLE 2:**
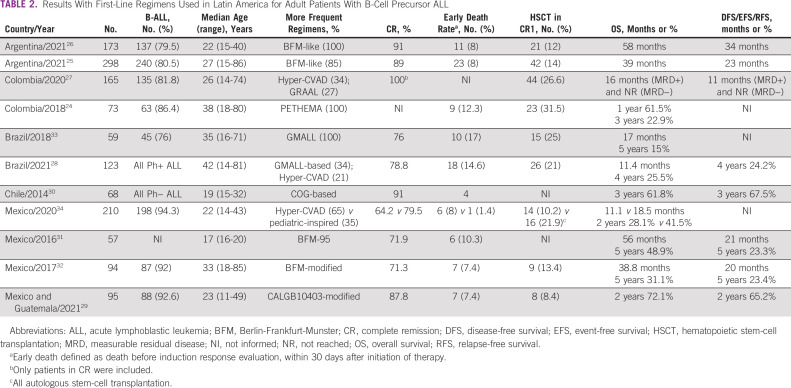
Results With First-Line Regimens Used in Latin America for Adult Patients With B-Cell Precursor ALL

### Adult Treatment Protocols ALL Ph–

#### Pediatric-inspired protocols: For eligible patients younger than 40 years.

Different prospective trials have validated the role of first-line treatment with pediatric-inspired protocols in adolescents and young adults. The primary treatment is continuous exposure to intensive and cumulative doses of nonmyelotoxic drugs such as asparaginase and corticosteroids. These protocols have early patient stratification per response during and/or after induction and consolidation, increasing chemotherapy doses when the therapeutic response is unfavorable. Administered hematopoietic growth factors avoid delaying the number of cycles.^1^ The most frequently used protocols are given in Tables 3 and 4. This panel recommends that any protocol such as GRAALL-2005 or Augmented BFM35^36^ may be used in young adults, according to each center's expertise.

**TABLE 3:**
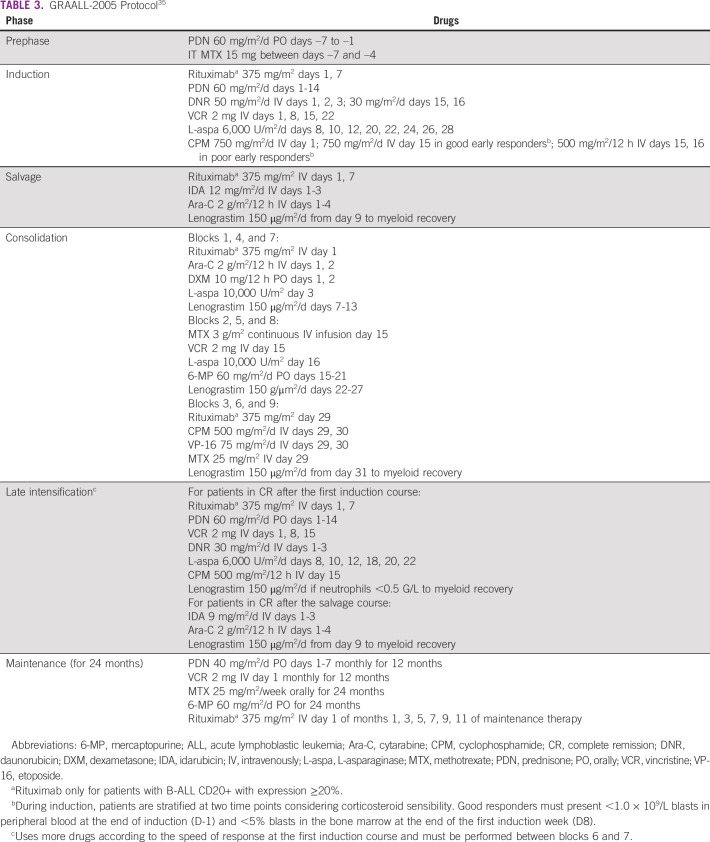
GRAALL-2005 Protocol^35^

**TABLE 4:**
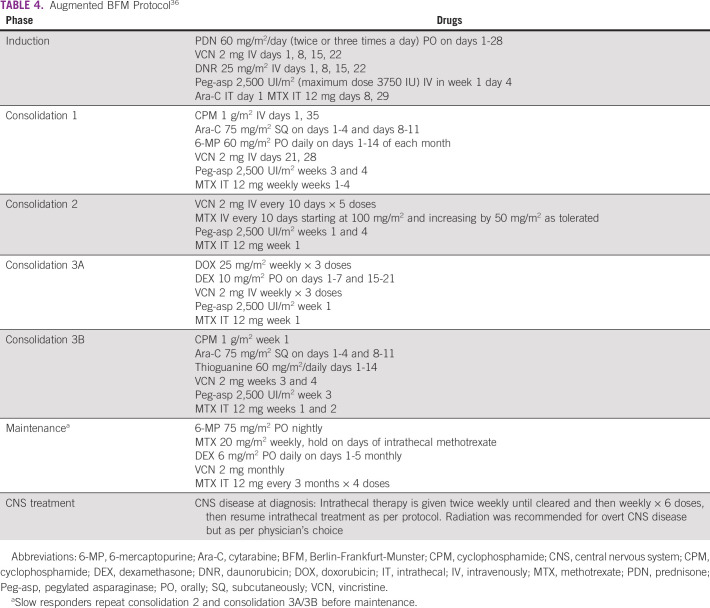
Augmented BFM Protocol^36^

#### Intensive treatment for patients older than 40 years but 60 years and younger.

Hyper-CVAD (Table 5) is an intensive protocol for patients older than 40 years yet younger than 60 years with appropriate organ fitness.^19,37^ The CR was 92%, and with a median follow-up of 63 months, OS and CR were 38% in 5 years.^38^ Hyper-CVAD is highly myelosuppressive and requires well-organized and funded departments. Early death rate and OS were worse compared with pediatric-inspired protocols in patients younger than 45 years in Mexican experience.^34^

**TABLE 5:**
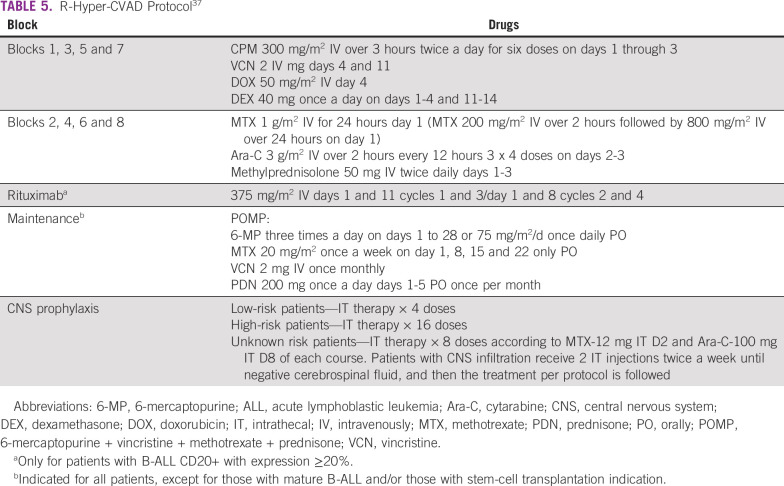
R-Hyper-CVAD Protocol^37^

In the CALGB-8811 (Table 6), patients were treated with a five-drug remission induction regimen. Those in CR also received consolidation treatment with multiple drugs followed by late intensification and maintenance for 24 months and CNS prophylaxis. The CR rate was 85%, and the mortality rate during induction was 9%. There were differences in CR rates according to age. Those younger than 30 years reached 94%, whereas those 60 years and older reached 39% (*P* < .001).^39^

**TABLE 6:**
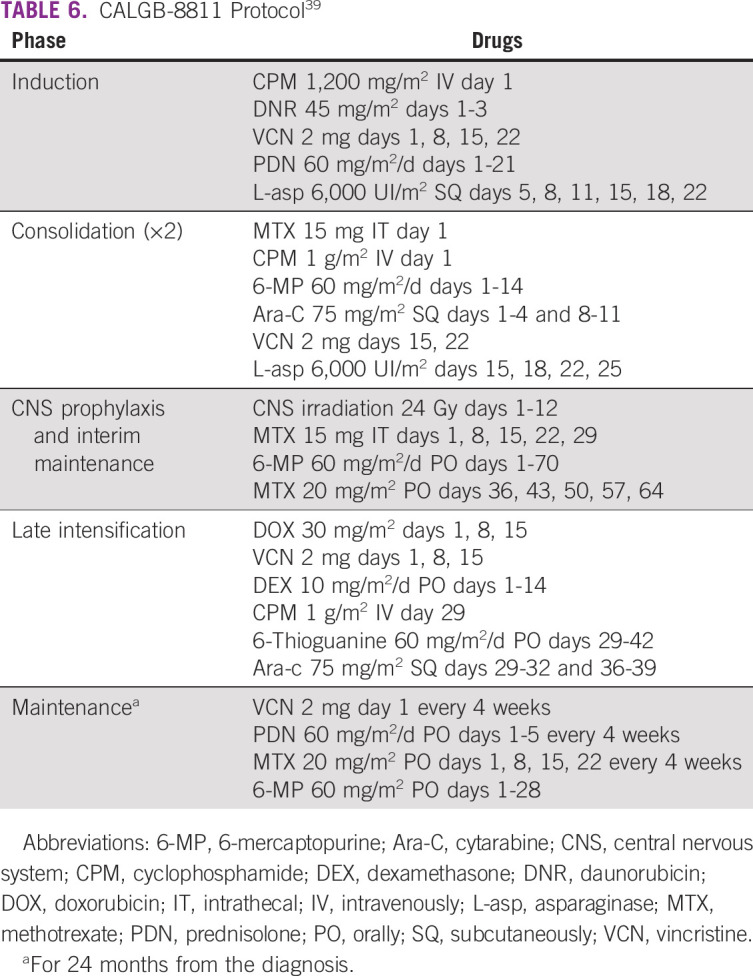
CALGB-8811 Protocol^39^

#### Treatment for patients older than 60 years.

When treating elderly patients, hematologists must consider treatment morbidity and mortality and eligibility for highly intensive consolidation therapies such as allo-HSCT, in addition to performance status. Treatment recommendations range from low-intensive protocols such as a combination of vincristine and corticosteroids or 6-mercaptopurine + vincristine + methotrexate + prednisone, whose objective is solely disease control, to moderate-intensity protocols on the basis of multiple drugs. Specifically for patients older than 60 years, the protocols must be adjusted using lower doses such as mini-Hyper-CVAD or GRAALL SA1.^40^ The aim is to reduce toxicity while striving for MRD− status.

### Ph+ ALL

Introduction of TKI represents one of the significant advances in the treatment of Ph+ patients, inducing deeper responses in combination with conventional chemotherapy. Imatinib and dasatinib are indicated in the initial treatment phases, and ponatinib is used in subsequent lines, especially in the case of resistance. It is essential to highlight that not all TKIs have been evaluated and/or compared with specific protocols. The TKI must be introduced at the induction phase and sustained throughout treatment until and after allo-HSCT.^41,42^ Combining imatinib with corticosteroids is feasible and can induce CR in most patients with Ph+ ALL without the need for hospitalization. Moreover, results from the EWALL-Ph-01 trial showed a 96% CR rate with dasatinib and low-intensity chemotherapy combination in elderly patients with high comorbidity scores and a 5-year OS of 36%.^43^

The CR from a recent phase 2 trial using dasatinib and blinatumomab, a chemotherapy-free protocol combination, as first-line treatment for Ph+ ALL was 98%. The median age was 54 years, and 38% of patients were referred to allo-HSCT.^44^

### Hematopoietic Stem-Cell Transplant for ALL

Adult induction regimens effectively induce CR, but maintaining remission and curing patients with ALL remains difficult. Long-term survival after relapse is low, and graft versus leukemia (GVL) can occur. allo-HSCT relies on chemotherapy/radiotherapy cytotoxicity and donor T-cell GVL.^45^

For SR patients in CR1 treated with pediatric-inspired regimens that achieve early MRD response, intensive chemotherapy consolidation and maintenance phases are recommended, with allo-HSCT reserved for CR2 patients. For HR patients and persistent MRD^46^ after induction, allo-HSCT as upfront consolidation is recommended. Patients with MRD+ disease may proceed with allo-HSCT with curative intent, but their outcomes are likely inferior to those with MRD− disease.^47^ In the case of MRD+ disease in patients without comorbidities, myeloablative conditioning (MAC) regimens should be prioritized.^45^

In Ph+ ALL, the added benefit of allo-HSCT in achieving deep molecular remissions with more potent TKI is now being questioned. Among patients treated with Hyper-CVAD + a TKI without allo-HSCT, the 4-year OS rate was 66% in patients who achieved complete molecular response at 3 months. By contrast, patients who do not achieve at least a major molecular response (MMR) may benefit from allo-HSCT in CR1.^48^ When imatinib was combined with Hyper-CVAD or a lower-intensity Hyper-CVAD in a randomized fashion, the benefit of allo-HSCT was restricted to patients who did not achieve MMR after two cycles.^49^ A retrospective analysis of patients with Ph+ ALL undergoing allo-HSCT in CR1 found that reduced-intensity conditioning (RIC) and MAC regimens were associated with similar overall outcomes for patients whose disease was MRD− before transplantation. By contrast, RIC was inferior in those whose disease was MRD+.^50^ Achieving BCR-ABL1–negative CR1 is likely favorable, but whether these patients can avoid allo-HSCT remains an area of active investigation. Therefore, allo-HSCT upfront for patients with Ph+ ALL is recommended.^45^

In patients with relapsed or refractory (R/R) ALL who achieve CR2, allo-HSCT should be performed.^51^ A positive MRD is associated with relapse, so the disease should be well-controlled before transplantation. Outcomes in patients undergoing allo-HSCT in CR2 are inferior to those in CR1. Patients with active disease should only receive allo-HSCT in a clinical trial.

### Conditioning Regimens for ALL

Randomized data supporting allo-HSCT for ALL come from older studies in younger patients treated with intensive MAC.^52,53^ However, no randomized data exist comparing RIC allo-HSCT with chemotherapy or MAC and RIC in patients with ALL undergoing allo-HSCT.^54,55^ A CIBMTR study of 1,521 patients with Ph− ALL receiving a MAC regimen (n = 1,428) or a RIC regimen (n = 93) found similar transplant-related mortality in the two groups but a greater risk of relapse in the RIC group, resulting in similar age-adjusted survival despite a substantially older median age. Similarly, another study by the European Society for Blood and Marrow Transplantation (EBMT) of 576 patients with ALL found that the RIC versus MAC was not significantly associated with leukemia-free survival (LFS), concluding that RIC allo-HSCT is a potential therapeutic option for patients not eligible for MAC.^55^

Reduced-intensive regimens make transplantation accessible to older adults with ALL. They have been associated with promising outcomes (3-year OS of 38% for all patients and 45% for patients in CR1, with no relapse occurring after 2 years) in patients older than 55 years .^54^ The current recommendation is to use MAC in fit patients and reserve RIC for patients not eligible for MAC. However, we acknowledge that other disease- and transplantation-related factors, such as MRD status, may influence the choice of conditioning intensity.^50^

Chemotherapy-based regimens have been studied to avoid short- and long-term toxicities of ablative total body irradiation (TBI). An EBMT analysis of 2,780 patients with ALL found that TBI improves LFS and OS over chemotherapy-based MAC, regardless of pretransplantation MRD status.^39-41^ Thus, we recommend TBI-based conditioning for patients deemed fit for MAC (until 40-45 years). Older or less fit patients should be treated with RIC regimens.

### Post–allo-HSCT Maintenance

Whether post–allo-HSCT maintenance therapy (initiated while the patient remains in CR) or pre-emptive therapy (triggered by the detection of MRD) improves disease control or survival remains unknown.^56^ Currently, there are no published reports of maintenance therapy for Ph− disease.

For Ph+ ALL, increasing evidence suggests that post–allo-HSCT TKI therapies targeting BCR-ABL may be associated with improved outcomes compared with historical data, including small prospective clinical investigations using imatinib, dasatinib, or nilotinib, as well as institutional experiences with various TKIs.^57^

Therefore, our current recommendation for patients with Ph+ ALL is to consider post-transplantation TKI as either maintenance or pre-emptive MRD-guided therapy although there are limited controlled data to support either choice.

### Targeted Therapies in ALL Treatment

The expression of CD20 in blasts of B-ALL has demonstrated an adverse prognosis and prompted the incorporation of rituximab, a chimeric MoAb targeting CD20, into intensive chemotherapy regimens.^58^ In a phase III trial, patients included in the GRAAL 2005 protocol were randomly assigned to receive chemotherapy with or without 16-18 rituximab infusions. When rituximab was given during all treatment phases, the estimated 2-year event-free survival rates were 65% in the rituximab group and 52% in the control arm (*P* = .04). Rituximab should probably be administered throughout the therapy, as the GRAAL trial did, since a study from UKALL investigators did not find any improvement when rituximab was given only in induction.^59^ Standard-dose rituximab incorporated into the Hyper-CVAD regimen improved outcomes for young adults with CD20+ ALL, with older patients not benefiting.^23^ Therefore, an anti-CD20 MoAb might be included in first-line treatment for adult patients with CD20+ (>20%) ALL.

Blinatumomab is a bispecific CD19-directed CD3 T-cell engager, fully approved by the US Food and Drug Administration (FDA) in 2017 for treating R/R ALL on the basis of two clinical trials.^60^ The treatment cycle consists of a 4-week continuous IV infusion, which is the main limitation of the drug's use. Neurologic side effects and cytokine release syndrome may deserve special attention when using the drug. In 2018, the FDA approved blinatumomab for patients in CR1 or CR2 with positive MRD disease.^22^

Inotuzumab ozogamicin (InO) is a humanized anti-CD22 MoAb conjugated to calicheamicin, a cytotoxic antibiotic. Inotuzumab ozogamicin is given as an intravenous infusion over one hour, making it more feasible in outpatient settings. Special attention concerning hepatotoxicity is necessary since this drug raises the risk of hepatic veno-occlusive disease or sinusoidal obstruction syndrome (VOD/SOS), mainly in older patients. Reducing the exposure to only two cycles can reduce the incidence of VOD/SOS.^61^

A phase 2 trial of mini-Hyper-CVAD in combination with InO as first-line treatment for older patients with ALL Ph− showed that 52 patients (median age of 68 years) had a 2-year progression-free survival of 59% and a mortality of 12%.^62^ Further studies are needed to consolidate the use of this strategy for older patients.

### Relapse and Refractory ALL

Treatment of R/R ALL represents an unmet medical need; new CR can be attained in 20%-40% of patients. The main reasons for treatment failure are refractory disease and relapse, and its prognosis remains poor.^63^ In adults, 5%-10% of patients are refractory to initial therapy and 30%-60% relapse despite improved CR rates and OS with optimized chemotherapy regimens and improvements in supportive care. The response rate decreases with successive salvage chemotherapy regimens, 21% after a second salvage and 11% after the third or higher salvage. The 1- and 3-year OS rates also decrease considerably after first (26% and 11%), second (18% and 6%), and third or higher salvage (15% and 4%).^64^ Predictors for outcomes include age, site of relapse, duration of the first remission, response to initial salvage therapy, disease burden at the time of diagnosis (particularly for patients with hyperleukocytosis), previous allo-HSCT, and fitness.

Multiple regimens have been explored to treat patients with relapsed ALL, but none has emerged as a standard of care. A regimen of fludarabine, high-dose cytarabine, and granulocyte colony-stimulating factor, either alone or in combination with idarubicin (FLAG-IDA), yields response rates around 40% in the relapsed and refractory setting.^65^

Blinatumomab, InO, and chimeric antigen receptor (CAR) T-cell therapy for R/R B-ALL has achieved CR2 with significantly improved response rate, including a proportion of undetectable MRD, altering the treatment landscape for these patients.^66^ Moreover, reduced toxicity allows patients to undergo allo-HSCT, serving as a bridge therapy to cure.

The phase III TOWER trial results, which compared salvage chemotherapy and blinatumomab in patients with Ph− ALL, had superior rates of CR (34% *v* 16%), 6-month event-free survival (4.0 *v* 7.3 months; HR, 0.55), and OS (7.7 *v* 4.4 months; HR, 0.71) in the blinatumomab arm, with 76% of MRD response for those in CR.^67^

The phase III trial InO-VATE that compared InO with standard-of-care chemotherapy in patients with R/R ALL demonstrated an overall response rate (ORR) significantly higher than the chemotherapy arm (36% *v* 17% and 81% *v* 29%, respectively).^68^ Responders had a higher rate of MRD negativity (78% *v* 28%), with more patients proceeding to allo-HSCT (41% *v* 11%).

Real-world outcomes of patients with ALL treated with InO are promising. For instance, in a multicenter retrospective study of 11 institutions across the United States, 84 patients who received InO outside of a clinical trial were evaluated. The ORR (CR/CR with incomplete count recovery) was 63%; 44% had CR with minimal residual disease negativity. Liver-related adverse events were comparable with the pivotal study,^69^ whereas the incidence of VOD was less than that previously reported.

In Ph+ ALL, the goal is to achieve negative molecular MRD. Patients with R/R Ph+ may be considered for second-line therapy with a TKI different from the one used as first-line. Mutation testing for the ABL/BCR is recommended, and the treatments are based on the mutation profile. Ponatinib has activity against T315I and effectively treats patients with R/R disease exposed to multiple TKIs. Combinations of TKI with multidrugs and/or MoAbs and/or corticosteroids should be an option for elderly patients who cannot tolerate intensive chemotherapy.^60^

### CD19 CAR-T Cells for ALL

CAR-T-cell therapy relies on the genetic manipulation of a patient's T cells to generate a response against a leukemic cell-surface antigen, most commonly CD19. Clinical trials using CAR T cells for B-ALL in adults showed a CR of 70%-89%.^70,71^ Two CAR T cells are approved for clinical use in the B-ALL–relapsed and B-ALL–refractory setting: tisagenlecleucel (4-1BB-CART19 product) on the basis of Eliana trial (25 years and younger) and brexucabtagene autoleucel (CD28-CART19 product) on the basis of the Zuma-3 study (adults 18 years or older).^53,72^ However, they are not yet in clinical practice in LA, except in Brazil where tisagenlecleucel has been recently approved.^73^

CAR-T-cell activation can cause severe cytokine release syndrome, immune effector cell-associated neurotoxicity syndrome (ICANS), and infectious risks, but treatment-related mortality is low and the side effects are usually reversible. Most experts favor consolidative HSCT after CAR-T, especially in HSCT-naïve and fit patients.^74^ The role of consolidative HSCT after CAR-T-cell therapy remains controversial.

### Panel’s Perspectives

**FIG 1:**
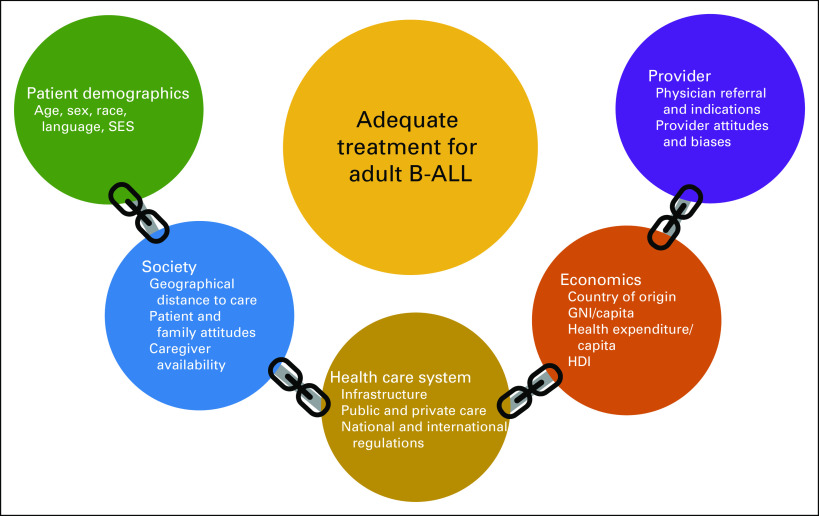
Barriers to adult B-ALL treatment in Latin America. ALL, acute lymphoblastic leukemia; GNI, gross national income; HDI, human development index; SES, socioeconomic status.

We must improve widespread and equal access to diagnosis, classification, and cutting-edge treatment for ALL. In this article, we have gathered the global information on adult ALL diagnosis and treatment and tried to adapt it to the realities of countries in LA. Different variables may affect the success of the ALL treatment. Figure 1 depicts the myriad barriers to access in LA, but unfortunately, these barriers have not been quantified to determine the contribution of each variable. One of the most critical areas is access to a high-quality and standardized MRD evaluation, identifying Ph+- and Ph−-like patients. There is a wide variation in transplant rates in the region, mostly related to GDP. Limited access to MoAbs, allo-HSCT,^8^ and CAR-T-cell therapy occurs in LA. These therapies also require specialized centers with trained personnel and regulatory approval.

Cost is the biggest limitation for implementing diagnostic tests and innovative treatments.^75^ Medication prices vary widely because of local negotiating policies. Lack of transparency regarding the prices that countries pay for the same drug exists. In some countries, government-led national funds have been established, but they are insufficient to increase access to medication. For instance, a panel of chronic myelogenous leukemia (CML) experts stated that the price of drugs reflects their unsustainable costs.^76^ On the basis of this analysis, we recommend forming a LA council comprising representatives from the pricing authorities of all nations. One of its goals would be to improve affordability for regional health care systems and manufacturers of leukemia medication. New policies for pharmaceutical companies would require full transparency of R&D costs, including company, public, and philanthropic investments. These could assess the cancer medicine's cost-effectiveness at various prices and incremental cost-effectiveness ratios per quality-adjusted life-year. Another approach to improve access established in Chile and Brazil is the Farmacia Popular Program, which provides a specified list of essential medicines subsidized by the government and supplied by many public and private pharmacies. This program has reduced drug reference prices by approximately 30%.^77^

Improved policies require a multistakeholder approach, including governments, the medical community and societies, academia, industry, payers, and patient advocates.

Each stakeholder has a different perspective regarding cancer care and provides unique viewpoints. By integrating data from these systems and industry sales, sophisticated data systems and metrics for measuring the quality of care and health outcomes could be used for concerted actions to establish priorities in cancer medication use and implement national interventions. Patient navigation, telemedicine, and artificial intelligence, which are increasingly used in high-income countries and show promising results, can partly alleviate health disparities.^78^ Up to 17% of patients with adult ALL had poor adherence to treatment in a Mexican study, and electronic health support has a great potential to improve access to health systems in this setting.^32^ There is also a need for clinical trials in LA. A consortium of medical professionals, similar to that seen for CML, across LA is needed for professionals dealing with ALL.

Finally, the publication of real-world data from cooperative groups working on ALL in LA helps us better understand the long-term benefit and cost-effectiveness of specific interventions. Every effort should be made to improve the outcomes in this patient population in all the countries of LA.
